# Supplementary material for: Are carbon emissions trading and green financial instruments synergistic? -Comprehensive quantitative research based on content analysis
Source: PLoS One. 2024 Mar 7;19(3):e0298601. doi: 10.1371/journal.pone.0298601 (PMC10919601; doi:10.1371/journal.pone.0298601)
Supplement: S1 Data — (ZIP) [file pone.0298601.s001.zip › Data/地级市Z_F工作报告.zip!/║╙─╧/╫ñ┬φ╡Ω╩╨/└┤╫╘╣1⁄2╓┌║┼í╛┬φ┐╦╩2╛▌═°í┐_2018.docx]

各位代表：

现在，我代表市人民政府，向大会报告工作，请予审议，并请各位政协委员和其他列席人士提出意见。

一、2017年工作回顾

2017年，面对复杂的经济形势和繁重的发展任务，在省委、省政府和市委的坚强领导下，全市上下以习近平新时代中国特色社会主义思想为指导，认真落实党中央、国务院和省委、省政府一系列决策部署，以推进供给侧结构性改革为主线，以打好“四大攻坚战”为抓手，综合施策、精准发力，保持了经济社会持续健康发展的良好态势，主要指标增速继续保持全省第一方阵，较好完成了市四届人大一次会议确定的各项目标任务，实现了本届政府良好开局。

——经济运行稳中向好。全市生产总值突破2000亿元，增长8.3%，规模以上工业增加值增长8.7%，均居全省第五位；全社会固定资产投资增长11.9%，社会消费品零售总额增长12.7%，均居全省第一位。

——质量效益不断提升。全市规模以上工业主营业务收入增长12.5%，实现利润增长19.3%；一般公共预算收入增长13.9%，居全省第四位，其中税收收入增长17%；节能减排完成省定目标，万元生产总值能耗下降1%以上。

——结构调整成效明显。三次产业结构达到18.7︰40.2︰41.1，二三产业比重提高1.6个百分点；全市服务业增加值增长10.4%，贡献率50.5%；秋季粮经比达到4︰6；高成长性制造业增加值增长12.7%，高新技术产业增加值增长13.2%；常住人口城镇化率达42.39%，提高1.5个百分点。

——人民生活持续改善。十件重点民生实事全面完成。完成全面改薄学校554所，困难群众大病医疗报销比例平均提高17.6%；全年优良天数240天，比上年增加23天，PM10、PM2.5分别下降12%、13%；城乡居民人均可支配收入增长8.9%，高于经济增速0.6个百分点。

一年来，重点抓了以下工作：

（一）抓产业促发展，经济保持稳定增长。大力发展工业经济。深入开展企业“降本增效”专项行动，切实搞好企业服务和各项政策落实，促进了工业经济持续稳定发展，全市规上工业增加值增长8.7%，高于年初目标0.3个百分点。深入推进工业项目建设，全年集中开工亿元以上重点项目119个，总投资576.4亿元，中原生态皮革产业园、今麦郎二期等重点项目开工建设，德威电缆、鹏辉锂电池二期等重点项目稳步推进。全市二星级产业集聚区达到2个、一星级8个，产业集聚区规上工业主营业务收入增长18.4%，规上工业增加值占全市的84.4%。积极发展现代农业。全市新建高标准粮田100万亩，全年粮食总产量132.5亿斤，为历史第二高产年。省级农业产业化集群发展到21个，总量居全省第一；市级以上重点龙头企业达到370家，农产品加工企业达到1500家。正阳县国家现代农业产业园正式获批，成为我省第一家国家级现代农业产业园。现代畜牧业持续发展，生猪、肉牛饲养量分别居全省第一、二位，肉蛋奶总产量分别达到71.8万吨、32.9万吨、3.85万吨。持续发展现代服务业。把服务业发展作为产业转型升级的重点，进一步完善措施，强化政策支持，加快发展步伐。全市服务业“两区”完成投资121.4亿元，建成区面积7.22平方公里，主营业务收入160亿元。全市房地产投资增长8%，商品房销售面积增长11.9%。全市金融机构各项存款余额2823亿元，增长11.6%，贷款余额1418.9亿元，增长15.6%；郑州银行驻马店分行及上蔡、新蔡农商行挂牌开业，兴业银行获批升格分行。全年实现旅游综合收入185亿元，增长15.5%。马庄铁路物流基地、恒兴物流二期、福汉兴公路物流港、驻马店B型保税中心等重点项目进展顺利，全市电子商务交易额达307亿元，增长30.4%。加快推进重点项目建设。纳入全省“5818”投资行动计划的315个项目完成投资910.6亿元，占年度计划的101%；2017年度中央预算内投资项目全部开工建设，完成投资19.2亿元，投资完成率达76.85%；33个省重点项目完成投资213.9亿元，占年度计划的126%；296个市重点项目完成投资989.9亿元，占年度计划的121%。

（二）抓改革扩开放，发展活力不断增强。全面深化改革。积极推进“放管服”改革，取消行政职权158项、各类证明186项。全面实施“三十五证合一”，各类市场主体增长18.8%。农村土地承包经营权确权颁证基本完成，城市管理综合执法体制改革顺利推进，市城市管理局挂牌成立。医药卫生、社会保障、文化、盐业管理等改革进展顺利。强力推进开放招商。全市实际到位省外资金262亿元，增长8.4%，增速居全省第二；实际利用外资3.99亿美元，增速居全省第三；完成进出口21.95亿元。成功举办第20届中国农加工洽谈会，我市签约亿元以上重点项目176个，总投资815.3亿元。大力实施创新驱动。积极实施大众创业万众创新行动计划，加快推进各类创新载体建设，设立了国家知识产权局专利审查协作驻马店工作站，全年实施科技创新项目193项，新增国家级星创天地1家，新认定高新技术企业27家、省级工程技术研究中心9家，新建院士工作站2家。

（三）抓建设强管理，统筹推进新型城镇化。以实施百城建设提质工程为抓手，以创建全国文明城市、国家卫生城市为目标，加强城市规划建设与管理，着力构建城乡一体化发展新格局。加快推进中心城区发展。实施百城建设提质项目573个，已开工410个、竣工133个，累计完成投资152亿元。42条主次干道维修和27条新建道路、薄山湖和板桥水库引水以及“一园两馆”、国际会展中心等重点项目加快推进。深入开展创文创卫工作，一年一举成功拿下全国文明城市、国家卫生城市两项国家级金字招牌，大大提振了广大党员干部和人民群众的士气。加快推进县城建设。各县都谋划实施了一批城市综合体、片区开发等重点项目，县城功能不断完善，吸纳集聚能力明显提升。以改善农村人居环境、实施重点镇建设示范工程为重点，启动实施了工业强镇、商贸重镇、旅游名镇和特色小镇建设。加快推进基础能力建设。周驻南和息邢高速、G328国道快速推进，12条组团连通工程和火车站综合改造项目加快推进，火车站新站房建成投用，安罗、许信高速开工建设，宿鸭湖清淤扩容项目前期工作基本完成，一批水利、能源、电力项目顺利实施。

（四）抓重点补短板，全力打好四大攻坚战。强力推进农村脱贫攻坚。强化扶贫对象精准管理，加大资金整合力度，加强贫困地区基础设施和公共服务设施建设，深入推进产业扶贫，累计投入财政资金38.1亿元，实施各类脱贫项目3001个，完成193个贫困村整村脱贫、9.15万贫困人口稳定脱贫和3214人易地搬迁任务，新蔡县顺利通过省第三方验收和省级核查验收。积极探索贫困家庭重度残疾人集中托养模式，得到国务院扶贫办、民政部、中残联和省委、省政府的充分肯定。扎实推进国企改革攻坚。全年完成9家国有商贸企业改制任务，安置补偿职工2317人；国有企业社会职能剥离和“三供一业”移交进展顺利，向属地政府移交改制企业36家。强力推进环境治理攻坚。深入实施蓝天、碧水、乡村清洁工程，全面推行“河长制”，扎实开展大气污染防治、水污染防治专项行动，大气污染防治工作受到省政府表彰奖励。启动实施转型发展攻坚。制订了全市转型发展攻坚工作方案，确定了食品加工、装备制造、生物医药等9个重点攻坚产业，组织实施了171个、总投资1120.3亿元的转型攻坚重点项目，经济转型发展呈现良好态势。

（五）抓民生增福祉，社会大局和谐稳定。坚持保基本、兜底线、建机制，持续加大投入力度，全年完成财政民生支出339.2亿元，增长16.6%，占一般公共预算支出的79.6%，十件重点民生实事全面完成。稳定扩大社会就业。全年城镇新增就业8.3万人，失业人员再就业3.2万人、困难人员就业1.38万人，分别完成目标任务的158%、161%和197%；新增农村劳动力转移就业6.05万人，完成省定目标的121%。不断完善社会保障体系。城乡居民基本养老、医疗保险参保人数分别达419.2万人、661万人；加强残疾人、城乡低保人员和孤儿等困难群体生活保障，新的补助标准全面执行。新开工棚改安置房3.78万套，基本建成2.25万套，超额完成省下达任务。加快发展社会事业。完成全面改薄学校554所，中心城区34所中小学新建、改扩建项目加快推进；积极推进普通高中培优工程，本科一批上线人数增长23.8%；职教园区完成投资6.42亿元，职业技术学院、省交通技校等项目加快推进。成功举办第四届产教融合发展战略国际论坛。健康驻马店建设扎实推进，城市公立医院改革全面推开，药品加成全部取消，市中心医院、市第二人民医院成功创建三级甲等医院。加强公共文化服务体系建设，完成700个行政村（社区）基层文化服务中心建设任务。全年完成交通项目投资72.9亿元，占年度计划的103.9%，其中新建农村公路1979公里。加强社会治理创新。认真落实安全生产责任，深化重点行业领域专项整治，各类生产经营性伤亡起数和死亡人数实现“双下降”；集中化解信访积案，维护群众合法权益，越级访、非法访明显下降；深入贯彻最严谨的标准、最严格的监管、最严厉的处罚、最严肃的问责“四个最严”，全市食品药品安全形势稳定向好；强化社会治安综合治理，严厉打击违法犯罪和暴恐活动，人民群众安全感、满意度持续提升。

一年来，我们自觉接受人大法律监督和工作监督，自觉接受政协民主监督和社会监督，认真办理人大代表建议和政协提案，支持民主党派、工商联和无党派人士参政议政。切实加强民主法制建设，全面推进依法行政，法治政府建设取得新进展。健全权力运行制约监督，强化行政监察和审计监督，持续治理懒政怠政为官不为，营造了风清气正的政治生态和发展环境。以创建全国文明城市为抓手，深入开展社会主义核心价值观学习教育，公民道德素质和社会文明程度明显提升。积极推进军民融合发展，加强国防教育、国防后备力量和人民防空建设，我市荣获河南省双拥模范城“五连冠”。民族宗教、外事侨务、统计、气象、人防、史志、红十字、对口援疆等工作取得新成绩。

各位代表，2017年是驻马店跨越发展成效显著的一年，也是全市人民深感自豪的一年，我们办成了许多过去想办而没有办成的大事，驻马店已站在了高质量跨越发展新的历史起点上，“四个驻马店”建设呈现光明前景，千年古驿站、全国文明城、国家卫生城、美丽驻马店正在中原大地昂首挺胸、阔步前行！这些成绩的取得，是全面落实党中央、国务院和省委、省政府决策部署的结果，是市委坚强领导的结果，是市人大、市政协大力支持的结果，是全市上下攻坚克难、不懈努力的结果。在此，我代表市人民政府，向全市广大工人、农民、知识分子、干部，向驻驻部队指战员、武警官兵、公安民警及社会各界人士，表示崇高的敬意和衷心的感谢！

在肯定成绩的同时，也要清醒认识到，当前我市经济社会发展中还存在一些突出矛盾和问题：一是经济总量小，结构不合理。全市GDP总量居全省第11位，人均分别是全国的54%、全省的69%。产业结构不优，二三产业分别比全国、全省低11.7和9.6个百分点。特别是我市还处于工业化初期，工业企业普遍规模小、实力弱，目前全市规模以上工业企业仅3597家，主营业务收入仅占全省总量的3.6%，每千人拥有企业只有9家，远低于全国19家、全省11家的平均水平。二是城镇化水平低，以城带乡能力弱。2017年，我市城镇化率分别比全国、全省低16.1和7.6个百分点，城市基础设施和服务功能不完善，产业支撑和人口集聚能力不足。县域经济发展滞后，2017年我省105个县市一般公共预算收入平均12.6亿元，我市8县平均7.7亿元，比全省平均水平低4.9亿元。三是民生欠账较多，脱贫攻坚任务繁重。城乡居民收入偏低，就业、教育、医疗等公共服务质量不高，农村基础设施和公共服务落后，人居环境问题突出。全市仍有6个国家或省级贫困县，有22.2万贫困人口尚未脱贫。四是各类人才缺乏，发展方式落后。人才引进培养机制不活，科技创新、企业管理、高级工匠、农村乡土人才缺乏。创新投入少，拥有自主知识产权的核心技术更少，2016年全市研发投入强度只有0.43%，不足全省的三分之一和全国的六分之一；全市高新技术企业64家，仅占全省的2.8%。五是能力素质不高，工作作风不实。一些政府工作人员工作作风、能力素质与新时代要求还有较大差距，缺乏敢想敢闯敢干的韧劲、干劲，特别是资本运作、经营城市的能力不足，一些机关干部为官不为、懒政怠政，项目落地难、群众办事难问题还不同程度存在。对此，我们一定要高度重视、直面问题、勇于担当、逐步解决。

二、2018年重点工作

2018年是贯彻党的十九大精神的开局之年，是决胜全面建成小康社会、实施“十三五”规划承上启下的关键一年，我们既处在发展环境深刻变化的机遇期，又处在深化改革加快转型的挑战期。从外部环境看，当前世界经济虽复苏缓慢曲折，但呈现企稳向好态势，我省加快建设经济强省、打造“三大高地”、实现“三大提升”正在稳步推进，为我市跨越发展营造了良好的外部环境。特别是中央农村工作会议和中央1号文件，围绕实施乡村振兴战略，出台了财政优先保障、公共服务优先安排等一系列政策措施，为我们农业大市发展带来重大机遇。从发展阶段看，目前我国经济已由高速度增长转向高质量发展阶段，但我市作为经济欠发达的中部农业大市，仍处于工业化初期阶段，城镇化水平低，经济实力较弱。我们必须迈出更大步子、付出更多努力，既要推动高质量发展，又要保持高速度增长，实现弯道超车、跨越发展，与全国全省一道步入全面小康，我们仍面临诸多挑战。从自身基础看，去年全市生产总值突破2000亿元大关，五年年均增长8.7%，规上工业五年年均增长10.5%，二三产业比重五年提高7.4个百分点，经济发展态势良好；近年来，相继引进了山东鲁花、恒都食品、君乐宝、德威电缆等一批知名企业，培育壮大了昊华骏化、天方药业、十三香等一批骨干企业，产业基础更加坚实；特别是成功创建全国文明城市和国家卫生城市，城市承载能力、群众文明素养大幅提升，创造了“敢于争先、团结拼搏、克难攻坚、善作善成”的创文创卫精神，凝聚了今后发展的强大精神力量，我们一定要顺势而为、乘势而上，进一步开创我市高质量跨越发展的新局面。

今年政府工作的总体要求是：全面贯彻党的十九大和中央省委经济工作会议精神，以习近平新时代中国特色社会主义思想为指导，坚定市委对经济工作的领导，坚持稳中求进总基调，坚持新发展理念和高质量发展根本方向，统筹推进“五位一体”总体布局和协调推进“四个全面”战略布局，坚持以供给侧结构性改革为主线，着力创造优势打好“四张牌”，着力打好“三大攻坚战”，着力实施乡村振兴战略，着力深化改革开放，着力优化发展环境，着力打造产业发达之城，推动经济社会又好又快发展，奋力谱写“四个驻马店”建设、决胜全面建成小康社会、实现驻马店高质量跨越发展新篇章。

今年经济社会发展主要预期目标是：生产总值增长8.5%左右，规模以上工业增加值增长9%左右，全社会固定资产投资增长12%，社会消费品零售总额增长11.5%，一般公共预算收入增长10%，城镇新增就业5万人以上，常住人口城镇化率提高1.5个百分点以上，居民消费价格涨幅控制在3%以内，城乡居民人均可支配收入增长与经济增长同步并略高于GDP增速，节能减排、生态环保等约束性指标完成省定目标任务。

围绕上述目标，重点抓好六个方面的工作：

（一）坚定不移做大做强产业，全力打造产业发达之城。坚持以产兴城、以产富民、产城融合，以产业规划为统领，以项目建设为抓手，全面实施产业强市行动，稳定一产、做强二产、提升三产，推动三次产业互动发展，加快构建现代产业体系，全力打造“产业发达之城”。

大力推动“兴工强市”，做大做强工业经济。以12个产业集聚区为依托，以年度投资614.7亿元的167个重点工业项目为抓手，制订出台产业强市实施意见及扶优扶强、招大引强、企业上市、创新升级（1＋N）方案，做大做强主导产业，拉长拉粗产业链条，促进工业经济加快发展，实现工业小市向工业大市转变。扶优扶强一批。制定完善扶持政策，加大对现有企业扶持力度，做大做强食品、装备制造、能源化工、服装、建材、生物医药等主导产业，促进产业集群化发展；积极扶持昊华骏化、十三香、中集华骏等重点企业发展，抓好昊华骏化醋酸乙醇、骏化发展20万吨DMF及华中正大、天方药业、华骏铸造搬迁等重点项目建设，加大对“30强、30高、30新”企业扶持力度，促进企业集团化发展，着力打造一批年销售收入超30亿元、50亿元和100亿元的骨干企业。推动上市一批。抓住国家对贫困地区企业上市开辟绿色通道机遇，积极推进企业上市培育三年行动计划，强化上市后备企业培育辅导，加快推进蓝天燃气、顺达科技、骏化发展、龙凤山牧业、花花牛在主板上市和一批企业在新三板、中原股权交易中心挂牌，力争今年主板上市企业2~3家、2020年达到10家。转型升级一批。抓住国家实施制造业核心竞争力三年行动和新一轮技术改造升级机遇，按照“一个产业、一套班子、一个方案、一抓到底”要求，全力推进食品、装备制造、医药、化工等产业转型发展行动计划，加快推进恒久机械、中集华骏灯塔工厂、确山建筑产业园、云创谷汽车零部件产业园、惠洁新型建材、爱民药业、伊克斯达废旧橡胶循环利用等创新升级项目，认真落实增品种、提品质、创品牌专项行动，大力实施大中型企业市级研发机构全覆盖工程，引导企业与国内外科研机构联姻，加大技术改造和新产品研发力度，大力推进精品制造、品牌制造，实现产业产品创新升级。招大引强一批。围绕我市主导产业和集群发展，瞄准国内外500强、行业龙头企业和优秀上市企业，练好内功、主动出击，敢于善于与大企名企攀亲结缘，成功引进一批龙头骨干企业，抓好德威光纤光缆、杭萧钢构等优质项目洽谈对接，力争早日签约落地。开工建设一批。加快推进鹏辉锂电池三期、驻马店绿色建筑产业园、顺达化工100万吨醋酸以及金鼎远大、平舆户外休闲产业园等一批重点项目前期工作，确保尽早开工建设。建成投产一批。对德威电缆、西平嫘祖服装城、正阳机械产业园、平兴食品机械产业园等一批在建重点项目，要加快建设进度，确保尽早建成投产。通过精准施策，力争今年二星级产业集聚区有3个以上，市级形成1个具有核心竞争力的千亿级产业集群和2个500亿级产业集群，县区级形成10个百亿级特色产业集群。

大力实施乡村振兴战略，推进农业农村现代化。按照“产业兴旺、生态宜居、乡风文明、治理有效、生活富裕”总要求，编制实施乡村振兴战略规划，推动实现农业强、农村美、农民富，加快农业大市向农业强市转变。抓好国际农产品加工产业园建设。抓住农业部批准我市建设中国（驻马店）国际农产品加工产业园的机遇，高起点规划、高标准建设，努力打造国内一流、世界领先、产业发达的农产品加工中心；以办好中国农加工洽谈会为契机，积极推进国际国内合作交流、展示展销，加快实施农产品交易所建设，努力打造国内一流的农产品贸易中心；加快正阳国家现代农业产业园建设，支持西平畜牧机械示范园、平舆芝麻产业科技创新中心建设，在有条件县区开展田园综合体试点。大力发展高效农业。坚持质量兴农、绿色兴农、品牌强农，加快由增产导向转向提质导向。按照粮食保障供给、口粮绝对安全的原则，加快推进860万亩粮食生产功能区和32万亩重要农产品生产保护区建设，确保粮食总产稳定在130亿斤以上。以“四优四化”为重点，以高效种养业结构调整为主线，减少效益低下的籽粒玉米种植面积，推动花生、白芝麻、食用菌、林果、蔬菜、夏南牛等特色产业发展，培育一批叫得响的知名品牌，力争优质专用小麦发展到450万亩、花生500万亩、白芝麻80万亩、生猪出栏780万头、肉牛出栏55万头。深入实施主要农作物生产全程机械化行动，确保农业综合机械化率在86%以上，切实做好秸秆禁烧和综合利用。大力发展“旅游＋”“生态＋”等农业新业态，加快发展休闲农业、观光农业、体验农业。培育壮大龙头企业。坚持用工业化理念经营农业，持续抓好农业产业化龙头企业发展，加大对君乐宝、今麦郎、恒都食品、花花牛、华英禽业等龙头企业扶持力度，促其不断做大做强。加快鲁花花生油、乐源观光牧场、夏南牛产业园、克明面业等重点项目建设，促其尽早建成投产。围绕我市农畜产品资源优势，着力引进一批国内外知名龙头企业，打造一批“全链条、全循环、高质量、高效益”的产业化集群，力争省级产业化集群达到25个、市级产业化集群达到40个。积极培育新型农业经营主体。进一步完善扶持政策，探索由政府发起成立乡村振兴战略基金，积极推行“龙头企业＋合作社＋农户”模式，总结推广正阳红旗合作社土地托管经验，积极发展适度规模经营，全年新增农民专业合作社1000家、家庭农场500家、现代农业产业联合体20家，土地规模经营面积达到400万亩。支持农机、农技、农资、防疫、检测等社会化服务组织发展，努力形成服务主体多元化、服务效率高效化、服务形式多样化的农业社会化服务体系。强化农业科技支撑。完善农业科技创新体系，加大农业科技投入力度，推动农业科技创新和科技成果转化。深入开展粮食作物高产创建活动，推进新品种更新换代，持续提升粮食综合生产能力。深入开展科技人员包村和送科技下乡活动，引入项目管理机制，推行政府购买服务，支持各类社会力量参与农业科技推广。加强农村专业人才队伍建设，创新培育引进使用机制，发挥科技人才支撑作用；依托职业技术学院、驻马店农校建立现代农民培训中心，培养有文化、懂技术、善经营、会管理的新型职业农民。加强“四美”乡村建设。按照环境美、田园美、村庄美、庭院美标准，全面推进“四美”乡村创建工程。加强乡村环卫设施和队伍建设，完成“村收集、乡转运、县处理”三级环卫体系建设；坚持政府主导、市场化运作，结合实施“厕所革命”，积极推进县乡村三级污水处理体系建设，支持遂平、确山、汝南、驿城等县区先行先试。实施农村人居环境整治三年行动，集中开展农村沟渠河塘综合整治，实施乡村绿化提升工程，改善村容村貌、路容路貌。加强“四好”农村公路建设，新建和改造提升农村公路500公里；加快安全饮水工程建设，解决好90个贫困村饮水安全问题。结合“三块地”改革，按照农民自愿、产业支撑、保障收入、配套完善的原则，在中心城区、产业集聚区、县城和乡镇所在地周边，开展合村并城、合村并镇试点，统筹规划建设集中安置区，稳妥推进农民集中居住。大力开展县乡村文明三级联创，积极推进文化惠民工程，促进乡风民风文明。

大力推动“三产兴市”，加快发展现代服务业。积极运用“互联网＋”新模式，在改造提升商业、餐饮、住宿等传统服务业的同时，推动现代服务业加快发展，实现服务业弱市向服务业强市转变。加快商务中心区和特色商业区建设。充分发挥“两区”的载体作用，进一步加大招商力度，着力引进一批投资规模大、带动能力强、业态模式新的重大项目，加快市商务中心区及万达城市综合体、绿地高铁新城、农产品商品交易所、皇家驿站、平舆上河城、新蔡西湖生态商务中心区等重点项目建设，全年完成“两区”建设投资190亿元。加快文化旅游业发展。充分利用我市山水生态、历史文化资源，加快推进全域旅游策划规划。组建驻马店文化旅游发展股份公司，设立文化旅游产业发展基金，推动旅游业转型发展。进一步理顺景区管理体制。围绕“吃住行游购娱”传统六要素和“商养学闲情奇”新兴六要素，深挖我市盘古、梁祝、嫘祖等文化内涵，组建专业创作和资本运作团队，推出一些有影响力的文化产业项目。支持遂平县争创国家旅游示范区，推进嵖岈山温泉小镇国家级旅游度假区、老乐山5A景区和白云山、天中山文化园4A景区创建工作。加快景区连通工程、杨靖宇纪念馆等重点项目建设，启动宿鸭湖环湖生态休闲经济带规划建设，强化宣传推介，着力打造“六山四湖”、“两馆一站”、“两城一寺”等精品景区和线路，力争全年旅游综合收入增长20%以上。加快现代物流业发展。完善物流业发展规划和扶持政策，重点发展铁路物流、公路物流、冷链物流、电商物流等。借助恒兴物流成为阿里巴巴·菜鸟、京东合作商的机遇，加快恒兴物流二期项目建设。积极推进马庄铁路物流、万邦农产品物流园、福汉兴公路物流港等重点项目建设，推动大型物流企业在我市设立分拨中心，努力打造全国重要的现代物流节点城市。加快现代金融业发展。全面完成县级农信社改组农商行工作，实现村镇银行县域全覆盖。加大招行引资力度，引进2~3家股份制银行在我市设立分支机构，引进一批金融租赁、基金、证券、信托、保险等多牌照金融机构，不断完善金融体系。加强金企对接，引导企业用好资本市场，支持实体经济发展。加快健康养老产业发展。围绕老年化群体多层次多样化需求，充分发挥我市山清、水秀、林茂优势，完善产业发展规划，引进养老知名品牌，加快老乐山银发壹族养生养老园、平舆祥和养老服务中心等项目建设，全力打造豫南健康养老基地。加快电子商务产业发展。大力扶持驻马店电子商务产业园发展，积极引进国内外知名电商来我市搭建平台，推进跨境电商发展；鼓励我市商贸、工业企业线上交易，积极推进农村电商发展，扶持发展一批电商村、淘宝村，打造我市电商产业发展新高地。促进房地产业健康发展。制订出台“三改”三年行动计划，积极推进棚户区、旧城区和城中村改造，新开工棚户区改造项目96个10.8万套。认真落实国家、省促进房地产业健康发展的政策措施，着力引进一批信誉高、实力强的国内知名房地产企业，不断提升住房品质。加快已拆迁群众安置房建设，推进保障性安居工程建设，加快培育房屋租赁市场，逐步构建多主体供给、多渠道保障、租购并举的住房体系。

大力实施产业强县富民计划，不断提升县域发展实力。把县域经济发展作为产业强市行动的重要引擎，支持各县以“三区”建设为载体，发展优势主导产业，壮大县域经济实力，建设产业发达县，努力形成1城9县优势突出、产业集聚、各具特色、竞相发展的良好格局。以产业集聚区建设为载体，围绕主导产业和集群发展招大引强，集中引进一批集群式重大项目，拉长拉粗产业链条，壮大产业集群规模，促进县域工业经济做大做强。以服务业“两区”建设为载体，加快建设一批功能完善、特色鲜明的城市综合体和商业街区，推动现代金融、电子商务、文化旅游等现代服务业发展，打造县域经济发展新的增长极。以现代农业示范园区建设为载体，积极引进和培育龙头企业发展，推进“龙头企业＋合作组织＋农户”模式创新，使农产品就地加工、农民就地转移，不断提高农业附加值和农民收入。

大力推进企业家队伍建设，提升企业家创造创业能力。企业家是市场经济的灵魂，是各类生产要素的整合者，是建设产业发达之城的主体力量。壮大企业家队伍。研究制定营造企业家健康成长环境、弘扬优秀企业家精神、更好发挥企业家作用的实施意见，鼓励和支持具有创造创业能力的企业家脱颖而出，逐步建设一支规模宏大、对党忠诚、诚信守法、善于创造、敢为人先、专业专心、治企有方、兴企有为，推动驻马店高质量跨越发展的企业家队伍。提升企业家素质。加大企业家培训力度，定期组织优秀企业家到党校（行政学院）培训，到国内外名校名企学习考察。开办驻马店企业家大讲堂，聘请国内外知名专家辅导授课，不断提升企业家创造创业水平。建立完善服务机制。建立政府与企业家沟通联系机制，将每月15日定为企业服务日，组织各级各部门围绕企业需求开展高效服务。建立完善务实管用的企业家服务机制，让企业家有苦有处诉、有难有人帮、有事有人办，不仅要关注企业家的税收，更要关心帮助解决企业家遇到的困难和问题，不讲条件为企业家提供优质高效服务。更好发挥企业家作用。建立优秀企业家表彰制度，营造企业家政治上有荣誉、社会上有地位的良好社会环境，营造企业家找政府办事廉洁高效、快捷舒心的营商环境，引爆企业家创业激情，激发企业家创造精神，促使企业家主动投身我市产业发展，决不能让企业家流血流汗又流泪，以良好的环境吸引更多人才来驻马店创业，不断发展壮大企业家队伍，努力形成大企业顶天立地、中小企业铺天盖地、创新企业占领制高地、企业活力欢天喜地的生动局面，真正把驻马店打造成创造的沃土、创业的乐园。

（二）强力推进开放招商，着力增强发展新活力。坚持把开放招商作为加快发展的有效捷径，在抓好现有企业发展壮大的同时，以更加开放的胸怀、更加优良的环境、更加有力的举措，大力实施开放招商，促使更多优质项目在天中大地落地生根、开花结果。

强力推进招商引资。研究制订招商引资专项行动方案，立足主导产业和产业集群发展，紧盯国内外知名企业、行业龙头企业、品牌上市企业，坚持请进来走出去相结合，推动招商引资向选商选资转变，着力招引一批有辐射力、带动力、影响力的重大项目。加强与万达、绿地、上汽、柳州五菱等知名企业的洽谈，组织专业团队跟踪对接，促使项目早日签约落地。积极拓展招商渠道，围绕长三角、珠三角和京津等区域，开展定向招商；主动与外来客商“攀亲结缘”，以良好环境推动以商招商；对重大招商项目主要领导亲自出马，当好项目市县区长和项目局长，开展大员招商；切实加强软硬环境建设，推动环境吸商；加强与各地商会协会的联系沟通，开展商会招商；鼓励在外工作及务工人员为家乡发展牵线搭桥，开展老乡引商；支持我市现有企业参与重大企业重组并购，积极与国内外知名企业嫁接联姻，推动股权招商、并购招商，努力实现招引一个企业、落地一个项目、带动一个产业、拉动一批就业、造福一方人民。

大力开展招才引智。制定出台人才强市实施意见，科学编制人才引进规划，启动人才公寓建设，以更优的政策引进人才、以更活的方式用好人才、以更好的平台留住人才，为各类人才提供良好的工作生活和创业环境。切实加强科技创新、经营管理、高级工匠、社会管理等人才的引进培养，依托黄淮学院建立产业创新发展研究院，柔性引进各类高端人才，真正做到寻觅人才求贤若渴、发现人才如获至宝、举荐人才不拘一格、聚天下英才而用之。加强企业与高等院校、科研院所合作平台建设，支持实施一批产学研项目，吸引推动一批科技成果在我市转化。

健全完善招商机制。加强招商引资队伍建设，整合招商力量，成立市招商引资服务中心，在广州、上海等地设立招商窗口，赋予驻京办、驻郑办招商引资职能，建立市县两级专业招商团队，统筹抓好招商引资工作。完善招商引资政策和工作流程，明确项目包装、信息发布、洽谈签约、规划引领、土地保障、手续办理、开工建设等各环节的责任，形成部门联动、高效审批、土地等项目的招商工作机制，实现“包装一批、签约一批、开工一批、在建一批、投产一批、储备一批”的良性循环，切实提高项目履约率、资金到位率和项目开工率。

积极推动全面开放。按照自贸区标准，全面实行市场准入负面清单制度，着力推进投资自由化、贸易便利化、监管法制化、服务高效化，构建统一开放、运转高效、公开透明、竞争有序的市场体系。加强开放平台建设，推进南阳海关、信阳商检在我市设立办事机构，加快驻马店保税物流中心建设。高质量办好第21届中国农加工洽谈会，实现会展、招商成果效益最大化。支持西平县办好2018全球纺织服装供应链大会。积极组织企业参加境内外重大招商贸易活动，支持企业开拓国际市场，推动花生机械、畜牧机械、优质农产品等产品出口。主动融入“一带一路”“三区一群”国家战略，积极对接中欧班列、新郑机场、明港机场，推进海铁联运、公铁联运、公路物流港无缝对接，推动开放招商向更高层次、更宽领域拓展，确保实际到位省外资金增长7%，实际利用外资增长3%，完成进出口增长10%。

（三）深入推进百城建设提质工程，着力提升城镇化水平。坚持以人为核心推进新型城镇化，探索生态、融合、适度、集约、高效的城镇化路子，突出高水平规划、高标准建设、高效率管理，坚持硬件与软件、新区与老区、地上与地下、宜业与宜居“四个统一”，做好以水润城、以绿荫城、以文化城、以业兴城“四篇文章”，着力提升城市品质和承载能力。

加快推进中心城区建设。一手抓老城区提升改造、一手抓新城区高品位建设，以实施年度投资274亿元的557个百城建设提质项目为抓手，不断完善城市功能，提升城市品位。加快完善城市规划。加强城市设计和专项规划编制，做好火车站东片区、小清河片区、高铁西片区控规修编和设计，完成城市风道、景观视廊、地下空间开发利用、城市双修等15个专项规划编制工作。严格按照规划进行建设，维护规划的严肃性，增强规划的执行力。加强功能片区开发建设。加快推进七大片区和城乡一体化示范区建设，加快推进火车站片区西广场、铁路东区域棚户区改造等项目建设，推动火车站东西片区一体联动发展。加强市政基础设施建设。启动实施文明大道、解放大道、中华大道等15条道路升级改造和兴业大道、东明路、蔡州大道等91条道路建设，加快推进重阳大道下穿高铁及开源大道、解放大道、交通路下穿京广铁路工程。积极推进国家“公交都市”和省公交优先示范城市创建工作，加快公交场站、公交智能化系统建设。完成板桥水库、薄山水库引水工程，加快推进第二水厂扩建及第三水厂建设。完善热力燃气、电力通信等配套设施，积极推进城市综合管廊建设，完成第二污水处理厂二期、第三污水处理厂二期和第四污水处理厂建设，开工建设第五、第六污水处理厂，加快推进生活建筑垃圾处理和27座垃圾中转站项目建设。加强生态水系建设。抓好河流水系治理及双高楼水库与薛庄河、五里河连通补源工程，加快推进生态水系连通、雨污分流工程，开工建设骏马河改造工程，稳步推进北部新区海绵城市示范区建设。加强绿化提升工程建设。按照创建森林城市、以绿荫城要求，着力提升绿化水平。重点抓好主要路段、公园绿化提升工程，加快推进小清河城市公园、练江河带状公园建设，开工建设开源河、朱氏河带状公园。启动实施一批特色文化主题公园及机关庭院绿化提升工程，确保中心城区新增绿化面积290万平方米，绿地率达39%、绿化覆盖率达45%，人均公园绿地面积达16平方米。加强城市精细化管理。认真落实《驻马店市城市市容和环境卫生管理条例》，明晰市容和环境卫生管理区域划分和行政处罚裁量标准，完善路长制、门前三包、网格管理、综合考评、督查奖惩等长效管理机制，切实加强市政公用设施、市容环境卫生、园林绿化、违法建设治理、交通秩序管理，全面提升城市规范化、精细化、智慧化、市场化管理水平，进一步提升创文创卫成果。

统筹推进城镇建设。把百城建设提质与文明城、卫生城、园林城、森林城等创建活动结合起来，切实加强城镇规划建设与管理。完善县城和乡村规划。建立健全县乡规划机构，积极推进市县规划展览馆、乡村规划展览室建设。按照“五规合一”要求和小城市建设标准，认真做好县城总体规划、专项规划、详细规划和城市设计，切实抓好乡镇规划和村庄建设规划，推进城乡规划全覆盖。加强城镇建设与管理。以产业集聚区和服务业“两区”为依托，积极推动二三产业融合发展，筑牢人口集聚的产业支撑，实现以产兴城、以城聚人、以人兴产、以产富民。立足提升县城品位和完善功能，重点实施一批基础设施、生态环保、公共服务项目。抓好竹沟红色小镇、嵖岈山风情小镇、关王庙豫剧小镇、梁祝文旅小镇以及胡庙、蚁蜂等特色旅游小镇开发建设。以创建国家卫生城、全国文明城为目标，深入开展“文明美丽县城建设三年行动计划”，不断提升城市建设管理水平和市民文明素质。

切实加强基础能力建设。基础能力建设是拉动投资增长、推进城乡发展的重要支撑，必须抢抓政策机遇，搞好项目融资，加快实施一批重大基础设施项目，提升支撑能力，厚植发展优势。交通方面，按照“一中心五组团”发展布局，加快推进12条组团连通工程等一批续建项目建设。积极做好陇盐铁路（南驻阜段）、南—驻—周—商高铁等项目谋划争取工作；加快推进周驻南、息邢、许信、安罗高速和G328、S223等一批干线公路项目建设，开工建设107国道绕城项目，启动107国道向东改线前期工作；加快推进嵖岈山通用机场前期工作，开工建设平舆通用机场，努力打造驻马店交通新优势。水利方面，开工建设宿鸭湖水库清淤扩容工程，抓好板桥水库除险加固、中小河流治理、高效节水灌溉等项目建设，加快推进南水北调4县供水工程，加快推进出山店水库向正阳县、平舆县、上蔡县供水及洪汝河连通、淮河流域滞洪区、汝河治理、杨庄滞洪区改水库、平原洼地治理、大型灌区续建配套等前期工作。能源方面，抓好中心城区、县城和产业集聚区集中供热及87个乡镇天然气利用工程，协调推动青海—河南特高压直流工程换流站建设，抓好第三座500千伏变电站前期工作，开工建设220千伏电网项目4个、110千伏18个，完成上蔡、平舆、确山3个贫困县及25个深度贫困村电网升级改造，实施好光伏发电扶贫项目和风电等新能源项目建设。信息化方面，加快数字化城市管理平台建设，实施好城市基础网络完善、宽带乡村等重大工程，加快大数据、云计算、物联网、现代通信基础设施建设，努力打造智慧城市。

（四）切实优化营商环境，着力增强创造创业新动能。进一步深化改革、提升效能、强化服务，营造审批更少、流程更优、效率更高、服务更好的营商环境，着力增强创新创业新动能。

全面深化改革。深化“放管服”改革，纵深推进“三十五证合一”改革，全面推行市场准入负面清单制度，深入开展“证照分离”改革试点；完善信用监管体系，推进信用记录归集共享，坚决严惩失信行为，营造诚实守信、公平竞争的良好氛围。深化资本市场机制改革，强化资源资产化、资产证券化和经营城市理念，完善政府投融资体系，组建豫资公司驻马店分公司、金融控股公司、产业发展投资公司，设立产业发展基金，强化金融服务，助力产业发展，推动新型城镇化。深化市属国有企业和国有控股企业去行政化、去平台化改革，制定出台市属国有企业领导人员管理办法，建立完善法人治理机构，让平台公司真正成为独立的法人实体，按照现代企业制度运营。完善市土地储备中心职能，组建驻马店市土投公司，盘活土地资源，实现土地收益最大化。积极推进土地利用综合改革，完善承包地“三权”分置制度，落实集体所有权、稳定农户承包权、放活土地经营权；深化农村集体产权制度改革，探索盘活闲置农户和宅基地的办法，促进资源变资产、资金变股金、农民变股东，激活乡村沉睡资源，释放农村改革红利。持续深化行政执法、医药卫生、财税、科技、教育、文化等体制改革，为创新创业提供良好政务环境。

提升服务效能。完善便民服务中心功能，按照“凡批必进、进必授权、依法高效、廉洁便民”的原则，积极推进行政服务体制机制改革，把行政许可流程进行科学再造，倒逼各级各部门简政放权、优化服务，收回行政审批多余之手，伸长行政服务有效之手。研究制定深化放管服改革、提高行政效能的意见，全面推进行政审批事项在线办理，完善“一次告知、一窗受理、一次办妥”机制，宁可我们跑断腿也不让企业多跑一趟。强化企业至上理念，构建“亲”“清”政商关系，急企业所急、想企业所想，真正把企业的事当作自己的事来办。切实抓好市长热线平台建设，认真收集和办理人民群众的意见建议。持续开展“降本增效”专项行动，实施涉企收费目录清单管理，打好减税、降费、降低成本“组合拳”，切实减轻企业负担。建立完善部门服务企业考评机制，千方百计解企业之忧、解群众之忧。依法依纪从严查处各种吃拿卡要、懒政怠政行为，谁砸企业的饭碗，我们就砸谁的饭碗；谁让群众不好过，我们就让谁不好过，以务实高效的作风营造良好环境，推动高质量跨越发展。

实施创新驱动。加强与知名科研院所合作，制定出台加快科技成果转化的实施意见，进一步完善政策措施，吸引各类科技成果在驻马店转化。积极推进经济开发区、市产业集聚区组团申报创建国家级高新区。依托黄淮学院积极创建国家级大学科技园，建好科技成果孵化器，办好第五届产教融合发展战略国际论坛，推动校企合作、产教融合。新建一批省级以上企业技术中心、工程技术研究中心，争取一批省级创新平台晋升国家级，确保今年新增高新技术企业15家以上，组建产业技术创新战略联盟1个，实施省市级重大科技专项5个以上。

推进大众创业万众创新。认真落实扩大科研单位及科研人员项目经费管理自主权、下放科技成果“三权”、实行股权期权激励等政策措施，进一步激发科研人员创新创业积极性。支持和鼓励事业单位选派专业技术人员到企业挂职或参与项目建设，支持和鼓励事业单位专业技术人员兼职创新或在职创办企业、离岗创业，支持和鼓励事业单位设置创新型岗位。完善支持返乡创业政策措施，引导和扶持更多成功人士返乡创业。认真落实扶持创新创业各项政策，给予创新创业者更多的阳光雨露，着力营造人人想创新创业、人人敢创新创业、人人能创新创业的社会环境，努力形成政府鼓励创新创业、社会支持创新创业、政策扶持创新创业的良好局面。

（五）精心组织实施三大攻坚战，为全面建成小康社会奠定坚实基础。认真落实中央和省委、市委经济工作会议精神，坚决打好防范化解重大风险、精准脱贫、污染治理三大攻坚战。

打好防范化解重大风险攻坚战。强化底线思维，牢牢守住不发生区域性、系统性风险底线。稳妥化解企业风险。全面排查各行业企业风险，根据企业生产经营情况，实行一企一策、分类管控、加强帮扶；充分发挥市场配置资源的决定性作用，鼓励金融机构加快资产证券化步伐，拓展低成本资金来源，实现金企双赢；引导企业强化风险意识，加强财务管控，优化债务结构，降低债务杠杆，稳健有序发展。防控政府债务风险。摸清债务家底，找准问题症结，制订防范方案；严控债务增量，稳定化解存量，规范举债行为，查处各类违法违规行为。防范金融风险。突出降杠杆、治乱象、抓规范、强监管四个重点，做到早识别、早发现、早处置，堵住金融风险源头。切实加强信用体系建设，防范打击非法集资、金融犯罪，营造良好的金融生态。

打好精准脱贫攻坚战。围绕我市脱贫攻坚目标，深入实施大别山革命老区振兴发展规划，全力以赴、精准发力，确保泌阳、汝南、正阳省级贫困县如期摘帽，完成220个贫困村退出、10.8万农村贫困人口稳定脱贫和驿城区、确山县2518人易地扶贫搬迁任务。坚持精准方略。坚持把提高脱贫质量放在首位，真正把扶持对象、项目安排、资金使用、措施到户、因村派人、脱贫成效“六个精准”落到实处，做到脱贫工作务实、脱贫过程扎实、脱贫结果真实。突出产业扶贫。坚持把产业发展作为实现稳定脱贫的重点，围绕十大脱贫产业、九种带贫模式，通过调整农村种养结构、建设特色生产基地、培育新型经营主体、推动产业融合发展等方式，多渠道推进农村产业发展。扎实推进“百企帮百村”工作，发挥好企业在产业扶贫中的作用。抓好精准帮扶。细化教育扶贫、健康扶贫、金融扶贫、社会扶贫、兜底扶贫措施，整合扶贫资金，加强贫困村基础设施和公共服务设施建设，抓好“三通”及人居环境综合整治，确保贫困村生产生活条件有效改善。免费为5000户贫困重度残疾人家庭实施无障碍改造，实施精准康复家庭医生签约，推广上蔡县重度残疾贫困人员集中托养经验，实现所有乡镇贫困家庭重度残疾人集中托养全覆盖。积极推行平舆县互联网＋分级诊疗健康扶贫、泌阳县集体经济带贫、正阳县农业产业化扶贫等模式，加大对25个深度贫困村脱贫攻坚支持力度。激发内生动力。把扶贫与扶志扶智结合起来，把救急纾困与内生脱贫结合起来，加强教育引导、政策引导和典型引导，改进帮扶方式方法，建立正向激励机制，补齐精神短板，变输血为造血，实现可持续稳定脱贫。为确保脱贫攻坚各项工作落实，将2018年作为“脱贫攻坚作风建设年”，深入开展扶贫领域腐败和作风问题专项治理，切实解决作风不扎实、形式主义等问题，对弄虚作假、搞数字脱贫的要严肃查处。侯卫东官场笔记

打好污染防治攻坚战。实施蓝天、清水、净土行动，持续加大环境治理力度，全面完成省委、省政府下达的各项目标任务，不断满足人民日益增长的优美生态环境需要。深入推进大气污染防治。以打赢蓝天保卫战为重点，突出重点区域、重点企业、重点时段，狠抓以扬尘控制为重点的控尘、控排、控煤、控油、控烧、控黄标车“六控”措施落实，完成水泥等行业超低排放改造，引导企业加大环保投入，提高污染治理设施装备水平，力争达到高标准排放，确保全年优良天气在210天以上。持续实施水污染防治。坚持全域治理、系统治理、源头治理，认真落实“河长制”“湖长制”，坚决“堵住”排污口、污染源，系统推进水污染防治、水生态保护和水资源管理，完成涉及淮河、长江两大流域重点工程建设，持续推进集中式饮用水水源规范化建设。全面开展土壤污染防治。开展土壤污染综合防治先行区建设，加强土壤污染治理和农业面源污染防治，严格危险废物环境监管，有序搬迁和依法关停严重污染土壤的企业，努力守护一方净土。加快推进畜禽养殖废弃物资源化利用，资源化利用率在70%以上。加大生态保护修复力度。以建设良好生态体系为目标，加快生态保护红线、城镇开发边界控制划定工作，抓好西部山区、板桥和宿鸭湖水库、淮河正阳段等生态修复工程。建立健全市场化、多元化生态补偿机制，加快推进生态环境监测网络体系建设，完善控制污染物排放许可制，不断提升生态环境治理水平。全力推进国家森林城市创建工作。启动开展国土绿化驻马店行动，积极推进退耕还林、天然林保护、长江防护林工程建设，抓好农田防护林、三边（路边、河边、湖边）绿化、山区和平原“片林”绿化提升工程，加快金顶山、老乐山植物园建设，启动五里河湿地公园规划建设，确保国家森林城市创建成功。

（六）切实保障改善民生，着力增进人民福祉。践行以人民为中心的发展思想，加大民生投入，办好民生实事，进一步增强人民群众的获得感、幸福感、安全感。

办好群众急需普遍受益的民生实事。在全面完成省定民生实事的基础上，全力办好我市确定的10件民生实事。一是开工建设人才公寓800套；二是完成市县乡村四级就业服务体系建设任务；三是完成60所敬老院改造任务；四是完成城市社区卫生服务机构标准化建设任务；五是加强市县乡三级急危重症救治体系建设；六是完成370所学校农村义务教育全面改薄任务；七是完成中心城区24所中小学新建、迁建和改扩建任务；八是完成中心城区8个停车场建设任务；九是完成全市贫困家庭重度残疾人集中托养和日间照料项目建设任务；十是完成中心城区新增纯电动公交车160台、引进电动共享汽车100台任务。

着力扩大社会就业。坚持“抓产业带就业、抓创业促就业”，制定完善促进就业的政策措施，重点抓好高校毕业生、农村劳动力、就业困难群体就业工作，动态消除“零就业家庭”，确保全年新增城镇就业5万人以上。建立健全市县乡村四级就业服务体系，抓好各类就业岗位和适龄就业人员信息收集、技能培训、中介服务、就业管理工作。

完善社会保障体系。按照兜底线、织密网、建机制的原则，加强社会保障体系建设，确保城乡居民养老、医疗保险参保人数达到422万人和661万人。继续提高城乡低保、基础养老金、特困人员生活费标准，积极参与养老保险全国统筹。推动城乡居民基本医保与大病保险制度有效衔接，全面推行以按病种付费为主的多元复合式医保支付方式，探索将困难群众慢性病和重大疾病门诊诊疗费纳入基本医疗保险或大病补充保险报销范围。

大力发展社会事业。坚持教育优先发展，加快普及学前教育，全市新建幼儿园48所，改扩建幼儿园182所；认真实施义务教育“全面改薄”和“城镇扩容”规划，实现遂平、西平、驿城区、正阳、上蔡、确山、泌阳国家义务教育发展基本均衡县验收。推进高中扩容改造，不断提高高中办学质量；加快推进职教园区建设，抓好基础设施、入园学校项目建设。继续办好高等教育，支持黄淮学院转型发展。大力实施名校英才入驻计划，积极引进省内外名校到驻马店办学。积极参加省第13届运动会，承办好2018中国山地马拉松系列赛嵖岈山站比赛。加快推进健康驻马店建设，深入开展健康促进行动，扎实推进区域医疗中心建设，完善分级诊疗制度，提高家庭医生签约服务水平；坚持中西医发展并重，加快中医药产业培育；启动建设市妇幼保健中心、市疾病控制临床救治中心，加快推进市妇女儿童医院建设，力争市中医院儿科病房大楼建成投用；依托市中心医院和市中医院，筹建驻马店医疗集团，探索建立居民健康档案管理中心；深化爱国卫生运动，启动健康城市、健康村镇创建工作；加强乡镇卫生院发展，全面完成标准化村卫生室建设任务，完善市县乡村四级医疗体系。积极发展文化事业，抓好1000个基层综合性文化服务中心建设，完成250场戏曲惠民演出，加快蔡国故城等重点文物维修和文物科技保护中心项目建设。

推进社会治理创新。以“平安驻马店”建设为抓手，强化“安全隐患就是安全事故”意识，认真落实安全生产责任制，强化重点行业领域安全专项治理，排查清除各类安全隐患，确保实现“一个杜绝、三个持续下降”目标。推进食品安全城市创建，强化食品药品监管，对制假售假、制劣售劣行为依法严惩，让人民群众吃得更放心、用药更安全。推进突发事件预警信息发布体系建设，提升防灾减灾救灾能力。加强社会治安防控体系建设，深入开展为期三年的“扫黑除恶”专项斗争，依法打击各种违法犯罪和暴力恐怖活动。完善社会矛盾纠纷预防化解机制，积极推进社会心理服务体系建设，落实重大决策社会稳定风险评估机制，推行阳光信访、责任信访、法治信访，妥善解决人民群众合理诉求。扎实做好第九届村民委员会和第六届社区居民委员会换届选举工作。切实加强社区建设，全面提升社区管理水平。

加强国防教育、国防动员和国防后备力量建设，支持国防和军队改革，支持驻驻解放军、武警部队建设，抓好民兵和人民防空建设，深入开展双拥共建，推进军民融合发展。支持工会、共青团、妇联等人民团体发挥桥梁纽带作用，注重发挥工商联等社团作用。认真做好民族宗教、外事侨务、统计、档案、史志、气象、地震、援疆等工作。

三、切实加强政府自身建设

各位代表！人民对美好生活的向往就是我们的奋斗目标，我们将主动顺应人民群众新期待，切实把发展使命扛在肩上，把人民忧乐放在心上，把作风建设抓在手上，进一步强化服务、提升效能，努力造就信念过硬、政治过硬、责任过硬、能力过硬、作风过硬的干部队伍，着力打造高效政府、廉洁政府、服务型政府。

（一）加强政治建设。深入学习贯彻党的十九大精神，坚持以习近平新时代中国特色社会主义思想为指导，牢固树立“四个意识”，增强“四个自信”，自觉在政治立场、政治方向、政治原则、政治道路上，始终同以习近平同志为核心的党中央保持高度一致。深入推进“两学一做”学习教育常态化制度化，扎实开展“不忘初心、牢记使命”主题教育，切实加强政府系统思想政治建设，严守党的政治纪律和政治规矩，认真落实党中央和省委、省政府的决策部署，坚决维护市委总揽全局、协调各方的领导权威，始终与市委同心同德、同向发力，确保党的政令畅通、决策落地生根。

（二）推进依法行政。牢固树立法治思维，带头遵法学法、守法用法，提高法治素质和法治能力，着力营造办事依法、遇事找法、解决问题用法、化解矛盾靠法的法治氛围。完善政府议事规则和决策程序，落实法律顾问、经济顾问制度，做到依法、民主、科学决策。强化行政权力运行制约监督，自觉接受人大及其常委会的法律和工作监督，自觉接受政协民主监督和社会监督，高度重视民主党派、工商联和无党派人士的意见建议，认真办理人大议案、代表建议和政协提案。切实加大政务公开力度，让权力公开透明、阳光运行。深入推进服务型行政执法，严格公正文明执法，强化执法监督，做到法无授权不可为、法定职责必须为。

（三）狠抓工作落实。坚持把抓落实作为政府工作的永恒主题，以“马上办、落实好”为根本，大力弘扬“敢于争先、团结拼搏、克难攻坚、善作善成”的创文创卫精神，践行“行动迅速、善抓落实、勇争一流、廉政为民”的工作准则，以披荆斩棘、勇往直前、逢山开路、遇水架桥的韧劲，切实抓好各项工作落实，以一流的精神状态、一流的工作干劲、一流的工作业绩，向全市人民交一份满意的答卷。

（四）加强廉政建设。认真落实全面从严治党主体责任和“一岗双责”，坚持把纪律和规矩挺在前面，严格遵守廉洁自律准则、纪律处分条例等党纪党规，严格执行中央“八项规定”，驰而不息开展正风肃纪。坚持厉行节约，强化预算刚性约束，严控“三公”经费支出，把更多的资源和财力用在抓重点、补短板、强弱项上。加强对政府投资项目评审、工程招投标等关键环节的监督管理，严明财经纪律，强化审计监督。完善激励机制和容错纠错机制，加大对恶意诬告陷害他人行为的惩治力度，保护党员干部干事创业积极性。始终保持惩治腐败高压态势，加大对侵害群众利益的不正之风和腐败问题的惩治力度，以廉政建设和反腐败斗争的实效，保障发展、取信于民！

各位代表！迈步新时代，开启新征程，跨越发展时不我待，全面小康重任在肩。让我们紧密团结在以习近平同志为核心的党中央周围，在省委、省政府和市委的正确领导下，不忘初心、牢记使命，勇往直前、锐意进取，为决胜全面建成小康社会、实现驻马店高质量跨越发展、谱写驻马店更加出彩新篇章而努力奋斗！
